# Empowering trainees through understanding learning theory and changes in medical education

**DOI:** 10.12688/mep.19046.1

**Published:** 2022-04-25

**Authors:** Parag Singhal, Stephen Craig, Grace Boyd, Davinder Sandhu

**Affiliations:** 1 Internal Medicine, University Hospitals Bristol and Weston NHS Foundation Trust, Weston-super-Mare, Somerset, BS23 4TQ, UK; 2 Clinical Medicine, American University of Antigua, Coolidge, Antigua and Barbuda

**Keywords:** Trainee empowerment, medical education, learning theory, critical thinking, healthcare systems, FAIR principles.

## Abstract

**Background: **Empowering trainees to think critically about decision making should result in the National Health Service (NHS) being more efficient and cost effective, thereby reducing the wastage of precious NHS resources on unnecessary investigations, treatment, and consequently putting patients at risk. There is a major shift from acquiring knowledge to critical analysis and synthesis of information for decision making. Trainees must understand how healthcare systems function and consequences of their decisions on budgets and patient care. Equally, faculty need to appreciate that their role is changing from information provider to facilitator of learning through feedback and supervision, role modelling, and innovator of learning approaches.

**Methods: **A survey of 100 postgraduate trainees from the Severn Deanery was conducted on SurveyMonkey in March 2020 and January 2021.  The survey consisted of eight questions focusing on trainee responses to participation in clinical decision-making in the inpatient setting. An additional question on communication with the patient was included in the second iteration.

**Results: **With a response rate of 80, only 35% of trainees had their findings regularly verified by the consultant. One third of trainees reported that decisions were made by the consultant without asking their opinion on investigations or management. It was unusual for trainees to have any interaction with patients on consultant ward rounds and to understand the rationale for the requested investigations.

**Conclusion: **The poor consultant trainee interaction represents a serious lost opportunity for experiential learning with real time feedback. Training programmes should support trainees being given opportunities to nurture analytical, problem-solving skills, dealing with uncertainty among other attributes of patient management. Trainees need to become competent through the art of critical thinking and develop a professional identity. Through this they develop confidence and competence leading to better patient outcomes, and prevention of the depletion of healthcare budgets.

## Introduction

Currently, the UK National Health Service (NHS) along with other international healthcare systems function within a spiral of never-ending demand for high quality healthcare delivered efficiently, effectively, and all within a tight financial envelope. Key to this is the healthcare staff who must grapple with the seismic demographic changes as well as the impact of new technology and treatments. In addition, as the COVID-19 pandemic has shown, healthcare along with any other system is fraught with uncertainty, complexity, and chaos ^1^.

It is even more essential now with the COVID–19 pandemic how front-line staff are skilled and empowered with critical decisions making, which impacts on efficient use of limited resources and achieves better outcomes for patients ^2^. The Academy of the Royal Colleges in their publication ^3^ emphasise that up to 20% of all investigations and treatments have no benefit to patient care. The impact of the above study would suggest that a saving of over £1 billion a year could be realised across the NHS with a more judicious approach to clinical evidence and diagnostic testing ^4^.

**Figure 1.**
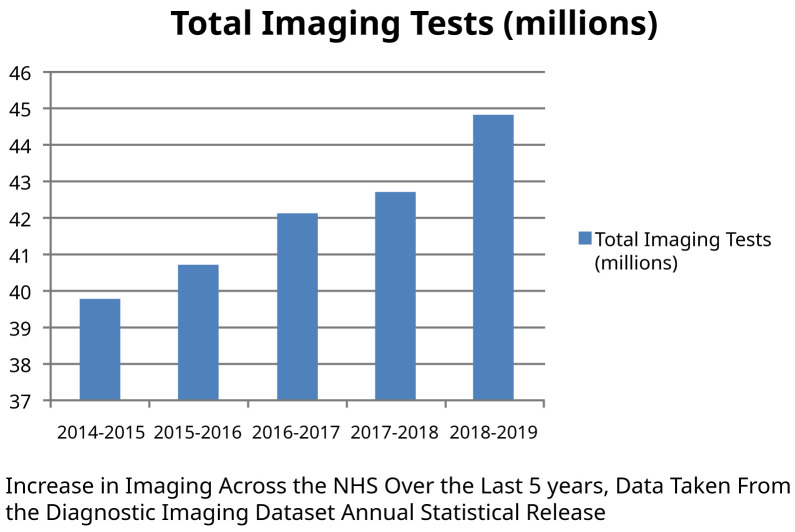
Diagnostic imaging dataset annual statistical release 2017/18. NHS England, 2018, demonstrating an exponential rise in CT and MRI scans ^6^.

Several reports have highlighted the fact that history taking and clinical examination is increasingly being replaced by investigations, both blood tests and diagnostic imaging without making a clinical diagnosis, along with widespread failure to adhere to the hospital diagnostic guidelines ^5^. This is confirmed by data that during recent years the NHS has seen an exponential rise in computerized tomography (CT) and magnetic resonance imaging (MRI) scans ( Figure 1) ^6^. The NHS audit commission have also reported that over £20 million a year is spent unnecessarily on inappropriate x-rays ^7^. The true number of inappropriate x-rays and CT/MR imaging is unknown, but this figure would match common consensus and opinion amongst physicians. If we consider that x-rays alone only make up a proportion of diagnostic requests, and the comparatively higher price of other imaging modalities, the overall financial burden of unnecessary radiological investigations is going to be much higher than this figure. The King’s Fund report submitted to the House of Commons has highlighted that despite significant improvement through innovation, the NHS still needs to increase value and productivity to reach the demands of an ageing and growing population in the UK ^8^. Elimination of unnecessary pathology and radiology tests by completing thorough patient examination and exercising critical thinking, could yield significant savings each year across the NHS which could be directly passed back into patient care ^8^.

NHS Improvement, the General Medical Council, and Health Education England have been arguing for daily consultant-led ward rounds, to enable early senior decision making to facilitate timely discharge thus reducing length of stay, and to provide closer supervision of trainees. The reduction in the length of stay has not happened for a variety of reasons, but mainly because of delayed transfers of patients with multiple co-morbidities and elderly patients staying in hospital, for non-medical/social reasons.

Although the consultant delivered approach would appear to confer safer working for junior doctors, but if trainees are not involved in understanding clinical thinking and patient management, trainees become disempowered to make independent decisions, and can lack confidence as new Consultants. Such training can lead to an over-reliance on investigations and a vicious cycle with new trainees using this as a management crutch, instead of making a judgement on the differential diagnosis through a critical examination of the traditional history and clinical examination.

## Methods

To gauge junior doctor’s perception of opportunities to take ownership and responsibility for decision-making an eight question survey on SurveyMonkey ( https://www.surveymonkey.com) of trainees (Foundation Year 1 to higher specialty trainees) was created by the authors and distributed by email to one hundred Severn Deanery trainees in two iterations of 50 trainees in each survey, from the trainee forum groups at the University Hospitals Bristol and Weston NHS Foundation Trust; North Bristol NHS Trust; Musgrove Park Hospital Trust; and Royal United Hospitals Bath NHS Foundation Trust. The survey was not piloted prior to distribution for this study. The survey was twice conducted, in 1–31 ^st^ March 2020 and in 1–31 ^st^ January 2021 and can be viewed in Table 1. No one particular group of trainees or hospital was targeted giving a good cross section of doctors in the Southwest of England. Targeting a hundred trainees would give a clear idea about the level of empowerment experienced by the trainees. There was no desire to seek any particular variables amongst the participants such as gender and age. The only variable was the level of trainees which have been captured by the demographic data from question one and discussed in the methods section. 

**Table 1.** Empowering trainees questionnaire.

**Question 1** What is your stage of training?					
FY1	FY2	CMT/IMT	ST3-4	ST5+	Non training post/ other please specify
**Question 2** How often do you see patients independently? (without a senior doctor / consultant?)					
daily	most days	sometimes	rarely	never	other please specify
**Question 3** If you see a patient independently, how often would a consultant validate your clinical findings?					
daily	most days	sometimes	rarely	never	
**Question 4** If your consultant requests an investigation for a patient, how often do they explain why?					
always	usually	sometimes	rarely	never	
**Question 5** How often do you feel that your opinion is asked for when reviewing patients with a consultant?					
always	usually	sometimes	rarely	never	
**Question 6** Are you involved in making decisions about investigations or prescribing treatments on a consultant ward round?					
always	usually	sometimes	rarely	never	
**Question 7** Are you involved in making decisions on investigations and treatments when reviewing a new patient on the medical take with a consultant?					
always	usually	sometimes	rarely	never	
**Question 8** Do you think examining patients for physical signs affects your medical management?					
always	usually	sometimes	rarely	never	
**Question 9** (asked on the second iteration of the survey) How often do you communicate directly with the patient on a consultant ward round?					
always	usually	sometimes	rarely	never	

From the experience of the first iteration of the survey in March 2020 an additional question (question nine) was asked in the second survey: ‘how often do you communicate directly with the patient on a consultant ward round?’ This question was added to seek further empowerment of trainee opportunities during the consultant ward round.

## Results

**Figure 2.**
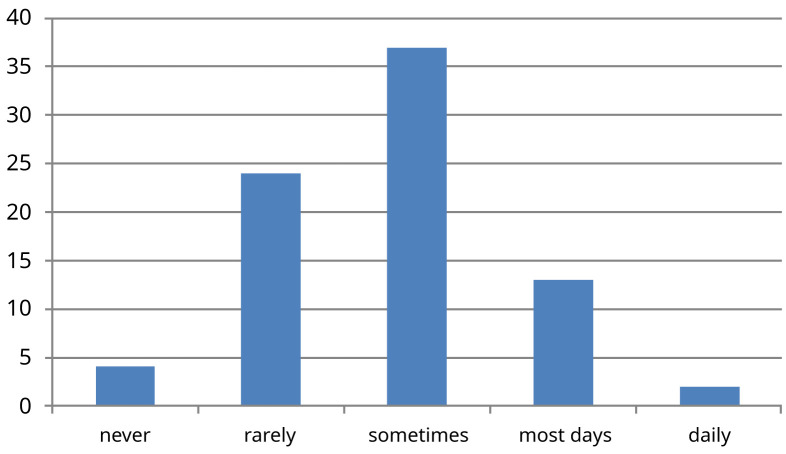
Results for survey question three ‘How often do you see patients with a consultant who validates your clinical examination findings?’ X-axis -absolute number of people who picked each response (n=80).

**Figure 3.**
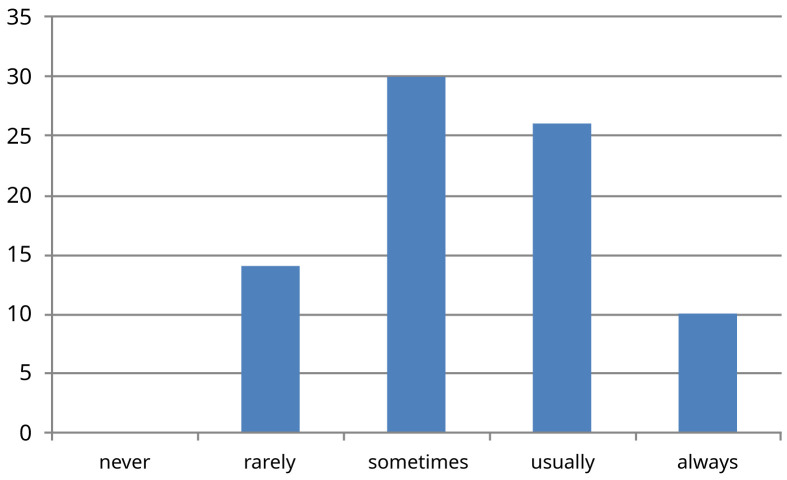
Results for survey question four ‘If asked to organise investigations for a patient how often are you clear on the rationale behind the investigation?’ X-axis -absolute number of people who picked each response (n=80).

**Figure 4.**
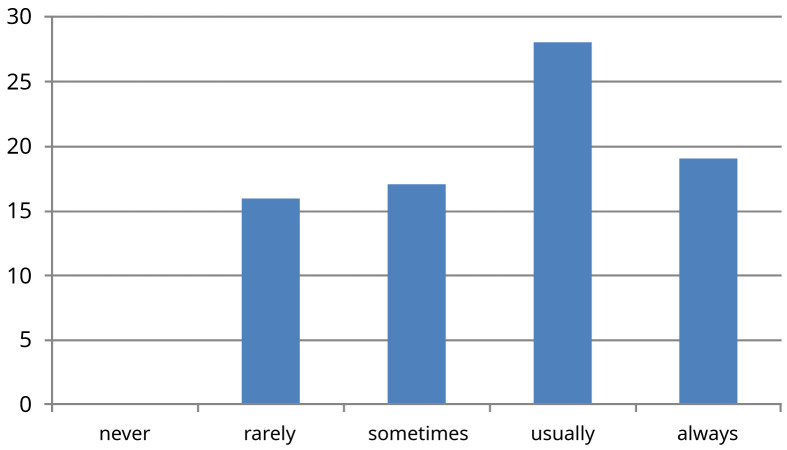
Results for survey question six ‘Are you involved in making decisions regarding ongoing patient management?’ X-axis -absolute number of people who picked each response (n=80).

**Figure 5.**
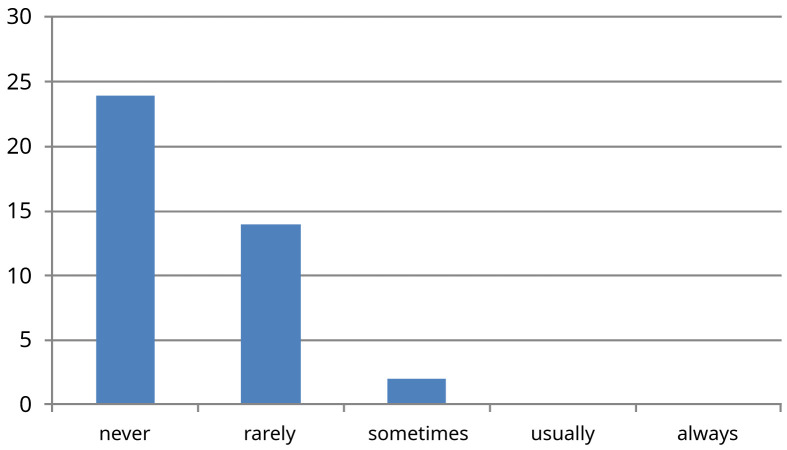
Results for survey question nine ‘How often do you communicate directly with the patient on a consultant ward round?’ X-axis -absolute number of people who picked each response (n=40) as this question was only asked in second iteration of survey as outlined in methods section.

The response rate was 80% (n=80) across both survey iterations, n=40 in March 2020 and n=40 in January 2021. No responses were excluded or incomplete. The demographic data from question one demonstrated 16 foundation year 1 doctors (20%), 44 senior house officers (SHO) and trust doctors grade (55%), and 20 specialist registrars (25%). While the majority of trainees (83%) of all levels reviewed patients independently daily (question two); approximately only 35% had their findings regularly verified by a consultant, (question three) ( Figure 2). 44% of trainees were not clear on the rationale for the investigations ordered (question four) ( Figure 3). One third of doctors surveyed said that usually decisions were made unilaterally by the consultant, without asking their opinion on investigations or management (question five) ( Figure 4). When asked if the trainees were involved in making decisions about investigations or prescribing treatment on a ward round (question six) 59% were involved, while 41% did so sometimes or rarely. ( Figure 5). As regards being involved in decision making when reviewing new patients with a consultant (question seven), 56% felt they were involved and 28% said sometimes, 10% rarely, and 5% never. Question eight explored the impact of physical signs in determining the management of patients. It is reassuring that an overwhelming 80% of trainees recognized the importance of examining for physical signs while 17.5% of trainees felt that such an examination only impacted their management sometimes, and 2.5% surprisingly said rarely. From the second iteration of the survey with the additional question, it was rare for trainees to have any interaction with patients during the Consultant ward round (question nine) as 60% said never, 35% rarely, and 5% said sometimes (n=40).

## Discussion

The failure of the consultant supervisor and trainee interaction represents lost opportunities to provide education with real time feedback on clinical examination and critical thinking ^9^.

### What needs to change?

An approach which empowers the trainees through taking provisional decisions with consultants validating their clinical findings, diagnosis, and management plans is essential. This should lead to improvements in team working, communication and leadership skills, empowerment, and confidence building ^10^. In practical terms, trainees should lead the early morning ward rounds and take decisions that are then validated by the subsequent consultant ward round and lead to an improvement in the morale of trainees and a sense of professional responsibility. 

As faculty and trainees within our epistemology, we need to understand and study what we see and experience to improve the quality of training and the care of patients. The Royal College of Physicians emphasises good clinical medicine which has a direct impact on patient safety. Clinical decisions are based on multiple skills such as pattern recognition, metacognition, ability to contextualise the patients’ needs, health beliefs, and risk perception amongst many other attributes. It is paramount in medical education to nurture high order critical thinking such as analytical and problem-solving skills, dealing with uncertainty or ambiguity, and the interpretation and application of small and big data. These trainees have to become change agents and come up with solutions for better, safer, cost-effective patient care.

### Understanding how learning occurs within a clinical context

Learning is not just content expertise or gaining new knowledge, but involves reflection, attitudes, and behaviours. Learning is a social process and occurs through interaction with each other through shared activities ^11^. There is no one time learning but a continuum of learning which is a social construct built through scaffolding knowledge learnt through our experience ^12^. However, the pressure of a medical career can create damaged learners. Trainees need to be trained in the art of translating patient care into the unique language, climate and characteristics of the medical setting. In the pressure of service delivery and defensive medicine, such entrustment is lost.

Trustworthiness therefore is a big part of clinical delegation. The complexity of making clinical decisions goes through three main phases. Initially there is clinical reasoning which is the thinking and decision-making processes associated with clinical practice. The next stage is clinical thinking which is based on constructivism ^12^ where previous knowledge, skills, observation, and judgement is built upon to inform patient management. And finally, critical thinking which consists of analysis of facts to form a judgement, ability to interpret an argument, evidence, or raw information in a logical and unbiased fashion, and thus solve complex problems effectively ^13, 14^.

Learning for trainees mainly happens during the acute take, exposure in emergency departments, outpatients and the Consultant ward rounds. Knowledge is contextually situated, and influenced by the activity, content, and culture in which it is used ^15^. The busy nature of medical practice often requires a need for rapid, senior led assessment and unilateral decision making. The bulk of clinical decisions are then made by the most senior and experienced member of the team. However, this approach has disempowered the trainees, especially the specialist registrars who are our future consultants. This urgency in management leads to a lack of educational discussion and the validation of clinical findings, with again reliance on expensive investigations and moving away from situated learning ^11^.

This means that trainees become invisible and are then dutifully delegated to complete request forms and referrals without the requirement for independent thought. There is little understanding of the process of how clinical decisions are made. Instead, trainees are driven by an unquestionable hierarchy and protocols which can stifle learning if they do not question the reasoning behind them and further, there is a failure to appreciate that each encounter is an educational encounter ^16^.

In addition, all of us must cope with the huge information explosion of over 60,000 diagnoses in medicine and more than 6,000 interventions ^17^. Despite this huge expansion in knowledge the time for training has not increased.

### Importance of clinical training

How junior doctors are trained will shape the future of the NHS in terms of their critical thinking skills and the need to reduce the burden of unnecessary investigations, treatments, and improve patient outcomes. 

This change in training must be profession led. Consultants and the wider NHS, for instance, need to be educated about the key educational pillars of feedback, activity, individualization, and relevance (FAIR) learning. 

Feedback is information communicated to the trainee that is intended to modify his or her thinking or behaviour in order to improve learning. The teaching ward round through real time feedback can correct mistakes, demonstrate physical signs, clarify goals, guide further studies, and reinforce good performance which are highly motivating. A trainee centred approach activates prior knowledge and new knowledge is built on to solve patient problems. The trainee is thus engaged in active rather than passive learning. Each trainee is an individual with a different level of mastery, learning preference, multiple intelligences, and personal capability ^18^. When all this comes together and is relevant to the problem in hand, then it is vastly rewarding and leads to deep learning. Consultants must take time to challenge trainees at the first point of assessment, on call, and on the medical ward rounds. Ward rounds must ensure that junior doctors are empowered and provided opportunity to review patients independently, make an assessment, and present their findings – right or wrong. They must then be engaged in discussion around their clinical findings, planned investigations, and their thought processes as the supervising consultant evaluates their decisions. This will provide positive reinforcement of correct findings and valuable lessons on areas for improvement. Consultants can lead on this by becoming positive role models ^17^.

## Conclusion

To improve their skills in critical thinking, trainees need to feel able to engage and challenge the consultants in discussion around clinical management and diagnostic tests. Consultants can then have confidence in their junior staff, and patients will have less needless investigation, intervention, and treatment such as inappropriate antibiotics which promotes the antimicrobial resistance pandemic ^19^. This should improve patient outcomes. Such cost-effective care will allow precious funds in the NHS to go a lot further and make the NHS much safer.

## Learning points

- Improve the quality of medical training and critical thinking skills of trainees.
- Give trainees the opportunity to problem-solve and make clinical decisions which are then validated by the supervising consultants.
- Raise awareness of the importance of correlating clinical examination and decisions with appropriate investigations.
- Reduce harm to patients from unnecessary or inappropriate investigations and treatment.
- Achieve major financial saving to the NHS from prevention of resources wastage.
- Consultants and education supervisors to be cognisant of the impact of role modelling and the FAIR principles.

## Data availability

 All data underlying the results are available as part of the article and no additional source data are required.
