# Supplementary material for: Empowering trainees through understanding learning theory and changes in medical education
Source: MedEdPublish (2016). 2022 Apr 25;12:30. [Version 1] doi: 10.12688/mep.19046.1 (PMC9370085; doi:10.12688/mep.19046.1)
Supplement: Peer review history

# Peer review: Reviewer response for version 1

**DOI:** 10.21956/mep.20404.r32028

**Published:** 2022-05-27

**Authors:** Andrew P. Day

**Affiliations:** 1 Department of Clinical Biochemistry, University Hospitals Bristol NHS Foundation Trust, Bristol, UK

The authors have observed that the ever increasing pressures on the Health Service have driven a consultant delivered approach to healthcare, with potential detrimental effects on the learning environment for trainees, and in particular their development of critical clinical decision making competencies. Lack of confidence in reliance on history and clinical examination in turn potentially leads to wasteful use of resources on expensive investigations.

 The authors have used a questionnaire to survey the experiences of medical trainees during consultant ward rounds. The results support the authors' hypothesis.

 The method used is clearly stated and the remarkably high response rate of 80% significantly reduces the risk of responder bias. The specialties of the trainees are not stated - were these all internal medicine?  It might be instructive to compare the responses in different specialties in the future.

 The results are clearly summarised, with some striking findings, particularly around how infrequently trainees participate actively in consultant ward rounds to the extent that they communicate directly with the patient rather than adopting a passive approach.

 As the authors correctly point out, failure to capitalise on the opportunities provided by an approach to consultant ward rounds that encourages the development of clinical problem solving skills and the recognition of the importance of clinical correlation is harmful to clinical training, with consequences for both patient safety and efficient use of limited financial resources.  

 Although consultant clinical and educational supervisors in the UK should all now receive training for these roles, the findings presented and discussed here suggest that further steps need to be taken to embed these principles, and health service managers need to be aware of potential adverse impacts of pushing for an ever more narrowly focussed consultant-provided service.

Have any limitations of the research been acknowledged?

Yes

Is the study design appropriate and does the work have academic merit?

Yes

Is the work clearly and accurately presented and does it cite the current literature?

Yes

If applicable, is the statistical analysis and its interpretation appropriate?

Not applicable

Are all the source data underlying the results available to ensure full reproducibility?

Yes

Are the conclusions drawn adequately supported by the results?

Yes

Are sufficient details of methods and analysis provided to allow replication by others?

Yes

Reviewer Expertise:

Medical education and assessment

I confirm that I have read this submission and believe that I have an appropriate level of expertise to confirm that it is of an acceptable scientific standard.

# Peer review: Reviewer response for version 1

**DOI:** 10.21956/mep.20404.r31929

**Published:** 2022-05-20

**Authors:** Jan Gutermuth

**Affiliations:** 1 Universitair Ziekenhuis Brussel (UZ Brussel), Department of Dermatology, SKIN research group, Vrije Universiteit Brussel (VUB), Brussels, Belgium

The manuscript shows that residency training does often not encourage own critical thinking by trainees. Moreover, residents often do not understand the rationale of orders from their attendings and often there is no room for explanation.

 The sample size is sufficient to draw the stated conclusions and the survey is clearly stated.

 This manuscript underlines the need to move the previous top-down approach in care processes in medical training centers, to a partnering approach between trainers and trainees.

 The recommendations from the authors have the potential to dramatically improve the training and problem-solving capabilities of future generations of physicians.

 Of note, current train-the-trainer programs (such as the compulsory training in Belgium) stress the need for supervisors to engage trainees in critical thinking and shared decision making between trainers and trainees.

Have any limitations of the research been acknowledged?

Yes

Is the study design appropriate and does the work have academic merit?

Yes

Is the work clearly and accurately presented and does it cite the current literature?

Yes

If applicable, is the statistical analysis and its interpretation appropriate?

Yes

Are all the source data underlying the results available to ensure full reproducibility?

Yes

Are the conclusions drawn adequately supported by the results?

Yes

Are sufficient details of methods and analysis provided to allow replication by others?

Yes

Reviewer Expertise:

healthcare delivery

I confirm that I have read this submission and believe that I have an appropriate level of expertise to confirm that it is of an acceptable scientific standard.
